# Planes de prescripción de actividad física y ejercicio en Atención Primaria en España: análisis comparativo autonómico

**DOI:** 10.1016/j.aprim.2025.103438

**Published:** 2026-04-27

**Authors:** Pedro Gutiérrez Moraño, Patricia Palomo López, Aron Carmelo Aredo Tisnado, María del Valle Ramírez Durán

**Affiliations:** a Servicio Extremeño de Salud, Gerencia del Área de Salud de Coria, Cáceres, España; b Universidad de Extremadura, Centro Universitario de Plasencia, Departamento de Enfermería, Cáceres, España; c Servicio de Salud de Castilla y León, Complejo Asistencial de Zamora, Zamora, España

**Keywords:** Atención Primaria de Salud, Terapia por ejercicio, Implementación de Planes de Salud, Servicios de Salud Comunitarios, Personal de enfermería, España, Primary Health Care, Exercise Therapy, Health Plan Implementation, Community Health Services, Nurses, Spain

## Abstract

**Objetivo:**

Analizar los planes vigentes de prescripción de actividad física y ejercicio en España, evaluando su grado de implementación, papel de la Atención Primaria, participación de enfermería y uso de recursos comunitarios.

**Diseño:**

Estudio descriptivo, transversal y observacional, basado en análisis documental de planes públicos, vigentes y oficiales de las Comunidades Autónomas.

**Emplazamiento:**

Ámbito nacional, en el contexto de Atención Primaria del Sistema Nacional de Salud.

**Participantes:**

Planes de prescripción de actividad física y ejercicio vigentes en las 17 regiones españolas y dos ciudades autónomas. Se incluyeron 11 planes oficiales; se excluyeron documentos no vigentes o con más de 15 años de antigüedad.

**Mediciones principales:**

Existencia de planes, inclusión de AP, rol de enfermería como prescriptor, incorporación de activos comunitarios, medidas de formación, divulgación, investigación y evaluación con indicadores.

**Resultados:**

Once Comunidades Autónomas disponen de planes activos; 10 contemplan la Atención Primaria, y 8 mencionan específicamente a enfermería como figura prescriptora. Se detectó alta variabilidad territorial, especialmente en medidas de evaluación (sólo ocho las incorporan) e investigación (sólo cuatro). Todas integran recursos comunitarios. Las medidas formativas y de colaboración intersectorial son frecuentes.

**Conclusiones:**

Existe una heterogeneidad significativa en el desarrollo e implementación de los planes de prescripción de actividad física y ejercicio. Se constata el papel clave de la enfermería en Atención Primaria y la necesidad de modelos coordinados con indicadores comunes, investigación aplicada y colaboración intersectorial para garantizar su efectividad y sostenibilidad.

## Abstract (en)

**Objective:**

To analyze current physical activity and exercise prescription plans in Spain, evaluating their level of implementation, the role of Primary Care, the involvement of nursing professionals, and the integration of community resources.

**Design:**

A descriptive, cross-sectional, observational study based on documentary analysis of public, current, and official plans from the Autonomous Communities.

**Setting:**

National scope, within the context of Primary Care in the Spanish National Health System.

**Participants:**

Physical activity and exercise prescription plans in force in Spain's 17 autonomous regions and 2 autonomous cities. A total of 11 official plans were included. Non-current documents or those over 15 years old were excluded.

**Main Outcome Measures:**

Existence of official plans, inclusion of Primary Care, recognition of nursing as prescribing professionals, incorporation of community assets, and presence of measures related to training, dissemination, research, and evaluation using indicators.

**Results:**

Eleven Autonomous Communities have active plans; ten explicitly include Primary Care, and eight specifically mention nurses as prescribing agents. A high degree of territorial variability was observed, particularly in evaluation (included in only 8 plans) and research (only 4). All plans integrate community resources. Training initiatives and intersectional collaboration measures are frequently included.

**Conclusions:**

There is significant heterogeneity in the development and implementation of physical activity and exercise prescription plans across regions. The key role of nursing within Primary Care is evident. Coordinated models with common indicators, applied research, and intersectional collaboration are essential to ensure their effectiveness and sustainability.

## Introducción

La práctica habitual de actividad física y ejercicio (AFyE) tiene importantes beneficios para la salud tanto en personas sanas como en personas con determinadas enfermedades crónicas, presentando aquellas que no hacen suficiente ejercicio físico un riesgo de mortalidad 20-30% superior a las más activas^1^, ^2^. Sin embargo, el 36,4% de la población de 15 años o más se declara sedentaria en su tiempo libre, siendo esta situación más prevalente entre las mujeres en personas con menor nivel educativo^3^. Este patrón de inactividad también representa un elevado coste para los sistemas sanitarios^4^. Ante esta situación, la Organización Mundial de la Salud (OMS) ha establecido como prioridad la promoción de sociedades más activas a través de su Plan de Acción Mundial sobre Actividad Física 2018–2030^5^. Esta estrategia insta a los gobiernos a implementar políticas que reduzcan el sedentarismo y aumenten la actividad física en todos los grupos de edad, contribuyendo además al cumplimiento de los Objetivos de Desarrollo Sostenible^6^. La Atención Primaria (AP), por su accesibilidad, cercanía y continuidad en la atención a lo largo del ciclo vital, representa el entorno idóneo para implementar planes de prescripción de actividad física y ejercicio (PPAFyE), adaptados a las características y necesidades individuales de cada paciente^7^. En este sentido, se ha demostrado que la intervención de los profesionales de enfermería de AP en la promoción del ejercicio puede aumentar significativamente la adherencia de los pacientes a las recomendaciones de actividad física^1^, ^8^, ^9^. Además, no perciben la falta de tiempo como una barrera con tanta frecuencia como los médicos en AP^10^, por lo que pueden actuar como agentes de cambio en la mejora de la salud de la población a través de la prescripción de AFyE adaptándolas a cada paciente y siendo una herramienta terapéutica, validada y supervisada, dentro del entorno clínico^11^.

El Ministerio de Sanidad ha impulsado iniciativas de diseño e implementación de PPAFyE en el marco del Plan de Recuperación, Transformación y Resiliencia, a través de su Componente 26: Fomento del Deporte 12, 13.

Existen Comunidades Autónomas (CCAA) que han desarrollado políticas autonómicas orientadas a la AFyE^6^. Sin embargo, un análisis reciente de Rial-Vázquez et al. (2023) señala que, aunque la mayoría de los planes de salud autonómicos incluyen medidas relacionadas con la promoción de la actividad física, estas requieren una revisión profunda debido al aumento de los comportamientos sedentarios^4^.

En este escenario, existe una gran brecha entre el conocimiento y su integración en la práctica clínica^14^, así como una falta de análisis específico sobre el grado de integración de la prescripción de ejercicio desde la AP, de la derivación a recursos comunitarios y activos de salud, aspectos clave para optimizar el impacto de estas estrategias.

Este estudio tiene como objetivo analizar la existencia de PPAFyE vigentes en España, comparando entre CCAA su grado de desarrollo e implementación, con especial énfasis en el ámbito de AP, sus profesionales como prescriptores de AfyE, así como en la integración de recursos comunitarios y activos de salud disponibles.

## Metodología

Se ha realizado un estudio descriptivo, transversal.

### Población

La población de estudio estuvo constituida por los diferentes planes y programas relacionados con la prescripción de actividad física y ejercicio en AP, implementados por las CCAA en España

### Criterios de inclusión

- Documentos técnicos como planes, estrategias, programas que incluyeran la prescripción de AFyE.
- Que estuvieran vigentes
- Que fueran de dominio público o publicados por parte de la administración.

### Criterios de exclusión:

- No se admitieron programas o planes de prescripción fuera de vigor o con una antigüedad superior a 15 años.

### Acceso a los datos

**Figura 1:**
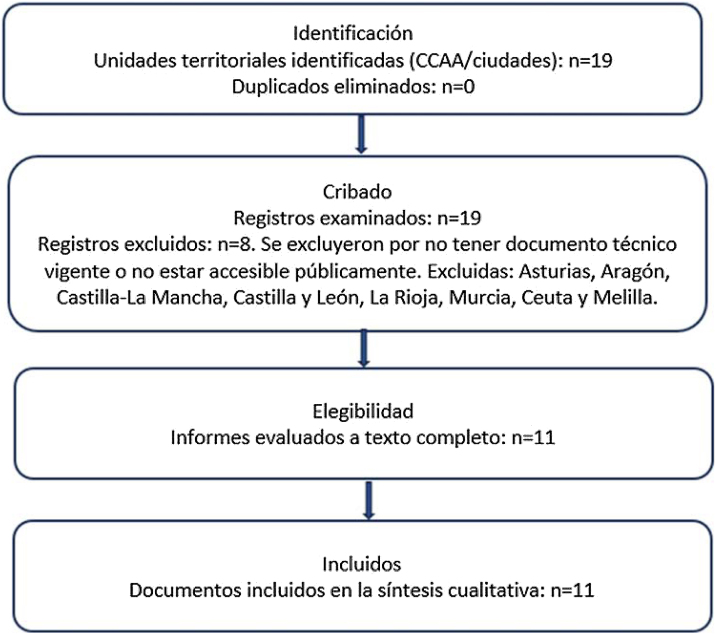
Diagrama de flujo de selección de PPAFyE (n = 19 unidades territoriales; incluidos=11; excluidos = 8 con motivos).

La búsqueda principal se realizó entre el 15 de noviembre de 2024 y el 31 enero de 2025 por dos investigadores, añadiendo una búsqueda con motivo de actualización previo a publicación del 1 al 20 de junio de 2025. La búsqueda abarcó documentos publicados entre enero de 2010 y junio de 2025, periodo correspondiente a la implantación progresiva de los PPAFyE en España. Se consultaron los sitios web institucionales de los 17 gobiernos autonómicos y sus consejerías de Sanidad, Deporte o Salud Pública; los Boletines y Diarios Oficiales autonómicos (BOJA, BOC, DOGV, DOE, BOCYL, BOPA, etc.), el Boletín Oficial del Estado y los portales de transparencia autonómicos. Además, se utilizaron buscadores web restringidos a dominios «.gob.es» y «.es», con combinaciones de términos como («prescripción de actividad física» OR «prescripción de ejercicio físico» OR «plan autonómico de actividad física») AND («Atención Primaria» OR «salud comunitaria») AND («CCAA»). Se realizó búsqueda manual en secciones de noticias y convocatorias de consejerías y contactos informales con responsables técnicos de Baleares, Castilla-La Mancha y Castilla y León. Todos los resultados se verificaron por duplicado mediante acceso a documentos oficiales (URL o PDF). Se contactó vía email con las Consejerías y Gerencias de las CCAA de Baleares, Castilla y León y Canarias. Véase el diagrama de flujo en la figura 1.

### Análisis de datos

La información relacionada con las variables de estudio de cada plan o programa de prescripción identificado se extrajo manualmente en una plantilla diseñada específicamente para este fin (respuesta dicotómica sí/no). Dos investigadores revisaron los documentos y las discrepancias se resolvieron en conjunto. Las variables analizadas se presentan en la tabla 1

**Tabla 1:** Variables de análisis y operativización

Código	Variable	Definición operativa	Criterio de codificación
V1	Existencia de documento técnico específico sobre la promoción o prescripción de actividad física y ejercicio	Documento oficial (plan, estrategia o programa) publicado o validado por una administración autonómica que incluya objetivos, medidas o líneas de acción en materia de actividad física y ejercicio como instrumento de salud	Sí: existe documento formal vigente con denominación y publicación oficial o institucional. No: inexistencia de documento específico o referencias genéricas en otros planes de salud
V2	Inclusión de la Atención Primaria como contexto de prescripción	Presencia explícita de la Atención Primaria (AP) como entorno donde se realiza la valoración, prescripción o derivación del paciente	Sí: se menciona AP o sus profesionales como ámbito de actuación o derivación. No: el plan se aplica solo al ámbito deportivo, educativo o comunitario
V3	Inclusión de profesionales de enfermería como agentes prescriptores	Mención explícita a enfermería en el texto del plan como profesional prescriptor o participante activo en el proceso de recomendación, valoración o seguimiento del ejercicio	Sí: cita literal de «enfermería», «personal de enfermería» o «profesionales sanitarios de AP incluyendo enfermería». No: no aparece o se refiere genéricamente a «profesionales sanitarios» sin especificar perfiles
V4	Integración de activos en salud y recursos comunitarios	Incorporación explícita de instalaciones, programas o entidades comunitarias (deportivas, municipales, sociales o educativas) como parte del circuito de prescripción o derivación	Sí: se mencionan recursos externos (polideportivos, asociaciones, rutas saludables, parques activos, etc.) o existe un mapa o catálogo de activos. No: el plan no prevé conexión con recursos comunitarios
V5	Medidas de intervención y gestión de recursos materiales e infraestructuras	Conjunto de acciones operativas o logísticas orientadas a implementar la prescripción de ejercicio o crear unidades, espacios o recursos para ello	Sí: se describen UAEF, circuitos de derivación, plataformas digitales o recursos materiales. No: solo recomendaciones teóricas sin medidas de ejecución
V6	Medidas de capacitación	Acciones formativas dirigidas a profesionales sanitarios o de actividad física y deporte relacionadas con la prescripción de ejercicio	Sí: formación reglada o específica (curso, jornada, módulo, etc.) prevista o implementada. No: no se contempla formación ni actualización
V7	Medidas de divulgación y colaboración	Estrategias de comunicación o cooperación entre sectores sanitario, deportivo, educativo o comunitario	Sí: se mencionan campañas, acuerdos intersectoriales o materiales divulgativos. No: no se describen acciones de comunicación ni cooperación
V8	Medidas de investigación	Inclusión de actividades orientadas a generar o difundir evidencia científica (recogida de datos, convenios con universidades, observatorios, publicaciones)	Sí: se mencionan estudios, evaluaciones con metodología científica o convenios académicos. No: ausencia de referencias a investigación o seguimiento analítico
V9	Evaluación con indicadores	Existencia de sistemas de evaluación explícitos con indicadores cualitativos o cuantitativos (rendimiento, impacto, adherencia, costes, etc.) y temporalidad definida	Sí: se especifican indicadores, plazos o informes periódicos. No: solo se menciona «evaluación» sin definir indicadores o cronograma

## Resultados

Un total de 11 CCAA cuentan con PPAFyE formalmente publicados de las 19 CCAA (57,9%) como se muestra en la tabla 2 con sus enlaces de acceso. Las regiones de Andalucía, Cantabria, Extremadura, País Vasco y Comunidad Valenciana, han publicado oficialmente planes autonómicos de prescripción de ejercicio físico en sus respectivos Boletines o Diarios Oficiales.

**Tabla 2:** Detalle de los PPAFyE y los enlaces a los documentos publicados

CCAA	Tipo de documento	Enlace de la publicación
Andalucía	Plan	https://www.juntadeandalucia.es/sites/default/files/2023-11/Plan%20Andaluz%20de%20Prescripción%20de%20Ejercicio%20Físico%202023-2030.pdf. Fecha acceso: 15/11/2024
Baleares	Plan	https://www.fundacioesportbalear.es/storage/app/uploads/public/64f/9a2/fba/Pla%20Promocio%20Act%20Fisica_v9.%2024_07_23.pdf. Fecha acceso: 10/06/2025
Canarias	Estrategia	https://www3.gobiernodecanarias.org/sanidad/scs/contenidoGenerico.jsp?idDocument=d427d5d9-ad64-11ef-baf9-273500fbdf02&idCarpeta=61e907e3-d473-11e9-9a19-e5198e027117. Fecha acceso: 20/06/2025
Cantabria	Resolución	https://boc.cantabria.es/boces/verAnuncioAction.do?idAnuBlob=385784. Fecha acceso: 20/11/2024
Cataluña	Guía	https://esport.gencat.cat/ca/arees_dactuacio/activitat-fisica-i-salut/2a-edicio-de-la-Guia-de-prescripcio-dexercici-fisic-per-a-la-salut/. Fecha acceso: 10/06/2025
Extremadura	Resolución	https://doe.juntaex.es/pdfs/doe/2023/2390o/23064197.pdf. Fecha acceso: 15/11/2024
Galicia	Plan	https://deporte.xunta.gal/sites/w_deport/files/documentacion/convocatorias/20241011_plan_de_prescripcion_de_actividad_fisica96.pdf. Fecha acceso: 10/06/2025
Madrid	Programa	https://www.comunidad.madrid/transparencia/informacion-institucional/planes-programas/programa-prescripcion-actividad-fisica-y-ejercicio-fisico. Fecha acceso: 15/11/2024
Navarra	Estrategia	https://www.navarra.es/nr/rdonlyres/94a0d714-6718-4bfd-b9b6-895d265bfb02/300857/prescripcionejerciciofisicoap3.pdf. Fecha acceso: 20/06/2025
País Vasco	Resolución	https://www.euskadi.eus/web01-bopv/es/p43aBOPVWebWar/VerParalelo.do?cd2023004598. Fecha acceso: 15/11/2024
Comunidad Valenciana	Resolución	https://dogv.gva.es/datos/2023/06/23/pdf/2023_6752.pdf. Fecha acceso: 20/11/2024

Por otro lado, Asturias y Baleares cuentan con leyes marco que regulan todo lo relativo al ejercicio físico y el deporte. Aragón y Murcia, a pesar de no disponer de un documento técnico publicado, sí disponen en la actualidad de un marco normativo para esta estrategia de salud pública a través de páginas web institucionales.

**Figura 2:**
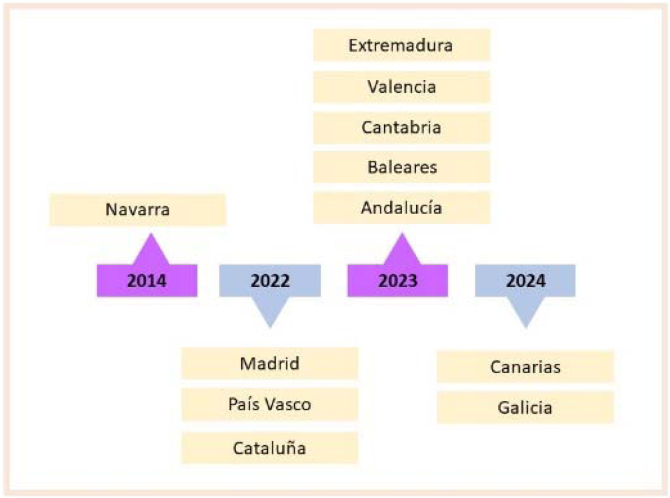
Cronograma de la publicación de planes y programas autonómicos de prescripción de actividad física y ejercicio.

Todos los PPAFyE analizados están en vigor, los años de publicación se muestran en la figura 2, estando la CCAA de Navarra en proceso de actualización en 2025.

La tabla 3 muestra de forma dicotómica el cumplimiento de las variables por parte de los PPAFyE y los porcentajes.

**Tabla 3:** Información recogida sobre los de PPAFyE existentes en las distintas CCAA

Comunidad Autónoma	V1	V2	V3	V4	V5	V6	V7	V8	V9
1.- Andalucía	Sí	Sí	Sí	Sí	Sí	Sí	Sí	Sí	Sí
2.- Aragón	No								
3.- Asturias (Principado de)	No								
4.- Baleares (Islas)	Sí	Sí	Sí	Sí	Sí	Sí	Sí	No	Sí
5.- Canarias	Sí	Sí	Sí	Sí	Sí	Sí	Sí	Sí	Sí
6.- Cantabria	Sí	Sí	Sí	Sí	Sí	Sí	Sí	No	Sí
7.- Castilla-La Mancha	No								
8.- Castilla y León	No								
9.- Cataluña	Sí	No	No	Sí	Sí	Sí	Sí	No	No
10.- Extremadura	Sí	Sí	Sí	Sí	Sí	No	Sí	Sí	Sí
11.- Galicia	Sí	Sí	Sí	Sí	Sí	Sí	Sí	No	Sí
12.- Madrid (Comunidad de)	Sí	Sí	Sí	Sí	Sí	Sí	Sí	Sí	Sí
13.- Murcia (Región de)	No								
14.- Navarra	Sí	Sí	No	Sí	Sí	No	Sí	No	No
15.- La Rioja	No								
16.- País Vasco (Euskadi)	Sí	Sí	Sí	Sí	Sí	Sí	Sí	No	Sí
17.-Comunidad Valenciana	Sí	Sí	No	Sí	Sí	Sí	Sí	No	No
18.- Ceuta	No								
19.- Melilla	No								
Porcentaje de cumplimiento	57,9% n (11)	90,9% n (10)	72,7% n (8)	100% n (11)	100% n (11)	90,9% n (9)	100% n (11)	36,4% n (4)	72,7% n (8)

V1: existencia o no de documento técnico específico sobre la promoción de AFyE en la CCAA; V2: se contempla la Atención Primaria como contexto de la prescripción; V3: se contempla a los profesionales de enfermería como agente prescriptor de actividad física y ejercicio; V4: se contempla los activos de salud y recursos comunitarios para mejorar la AFyE y reducir el sedentarismo; V5: medidas o estrategias de intervención y de gestión de recursos materiales e infraestructuras; V6: medidas o estrategias de capacitación; V7: medidas o estrategias de divulgación y colaboración; V8: medidas o estrategias de investigación; V9: indicadores de evaluación de las medidas adoptadas.

### Atención Primaria como contexto de prescripción, personal de prescripción de AFyE involucrado y activos en salud y recursos comunitarios

De los 11 PPAFyE analizados, diez contemplan la participación de AP (90,9%), siendo Cataluña la única excepción. En Andalucía, Cantabria, Galicia, Madrid, Canarias, Navarra y Comunidad Valenciana, la AP desempeña un papel activo en la prescripción de ejercicio desde las propias consultas. Todas, excepto Navarra (90,9%), disponen de Unidades Activas de Ejercicio Físico (UAEF) como estructura de apoyo. Baleares, Extremadura y País Vasco limitan la intervención de la AP a la captación y derivación de personas usuarias hacia las UAEF o programas específicos como «El Ejercicio te Cuida» en Extremadura, donde se realiza la prescripción. Madrid es la única comunidad que contempla una doble vía de prescripción: en AP y en el ámbito hospitalario. Por otra parte, Aragón y Murcia, que no cuentan con PPAFyE, mencionan en sus páginas web institucionales la derivación desde AP a unidades deportivas.

Respecto a las figuras prescriptoras, ocho planes incluyen específicamente a profesionales de enfermería (72,7%). Navarra y Comunidad Valenciana asignan esta función al equipo de AP sin precisar perfiles concretos, mientras que Cataluña, al no implicar a la AP, omite la definición de prescriptores. Las funciones atribuidas a enfermería incluyen cribado, valoración de la condición física y de salud, derivación, asesoramiento y seguimiento.

Todos los PPAFyE integran recursos comunitarios en sus estrategias, como instalaciones deportivas municipales, parques y escuelas deportivas. La organización de estos activos mediante mapas, guías o catálogos se observa en los planes de Cataluña, Madrid, Navarra y Comunidad Valenciana, y en plataformas digitales como «Localiza Salud» (Aragón) o «Activa Murcia».

### Medidas y estrategias desarrolladas por los planes y programas

Todos los PPAFyE analizados contemplan medidas de intervención, aunque con diferencias sustanciales en su alcance y diseño. Andalucía, Canarias, Cantabria, Galicia, Madrid y Comunidad Valenciana incluyen prescripción individual, grupal y comunitaria desde AP, así como la derivación a Unidades de Actividad y Ejercicio Físico (UAEF), considerando variables como el tipo de actividad física, su intensidad, duración y frecuencia. En Baleares, estas unidades asumen también la responsabilidad de prescripción. En Cataluña, se proponen recomendaciones generales e intervenciones adaptadas a variables sociodemográficas y clínicas, sin implicación directa de AP.

Extremadura plantea la captación desde AP y la derivación a técnicos deportivos encargados de implementar programas multicomponente (resistencia, fuerza, flexibilidad y equilibrio). Una lógica similar se observa en el País Vasco, donde la prescripción no se realiza en el contexto sanitario, pero sí se contempla la derivación a los Servicios de Orientación en Actividad Física.

Respecto a las medidas de capacitación, nueve (81,8%) incluyen acciones formativas dirigidas tanto al personal sanitario como a profesionales en ciencias de la actividad física y del deporte (CAFyD). Aunque los contenidos específicos no se detallan, sí se establecen duraciones concretas: Baleares prevé cuatro horas para sanitarios y País Vasco, cinco horas para sanitarios y 40 horas para profesionales CAFyD. Navarra y Extremadura no contemplan actividades formativas específicas. Madrid y País Vasco incorporan herramientas digitales con fines formativos, mientras que Andalucía subraya la formación del personal de enfermería como elemento estratégico para integrar la prescripción de ejercicio en los planes de cuidados.

Los 11 PPAFyE (100%) incluyen estrategias de divulgación y colaboración. Las acciones de sensibilización, dirigidas tanto a la población como a profesionales, se desarrollan mediante trípticos, campañas informativas, redes sociales y medios de comunicación. La colaboración interinstitucional se articula entre los ámbitos sanitario, deportivo y comunitario.

En relación con la investigación, Andalucía, Canarias, Extremadura y Madrid mencionan la recogida sistemática de datos para evaluar la efectividad de sus programas (36,4%). Destaca el caso de Extremadura, que mantiene un convenio con la Universidad de Extremadura mediante el Observatorio «El Ejercicio te Cuida», orientado a la generación y difusión de evidencia científica.

Por último, ocho planes incluyen medidas de evaluación y seguimiento (72,7%), siendo Cataluña, Navarra y Comunidad Valenciana las únicas que no lo hacen. Las metodologías varían, desde evaluaciones periódicas estructuradas por objetivos e indicadores específicos, hasta modelos más complejos como el de Canarias, que incorpora 39 áreas evaluativas supervisadas por un comité específico.

**Figura 3:**
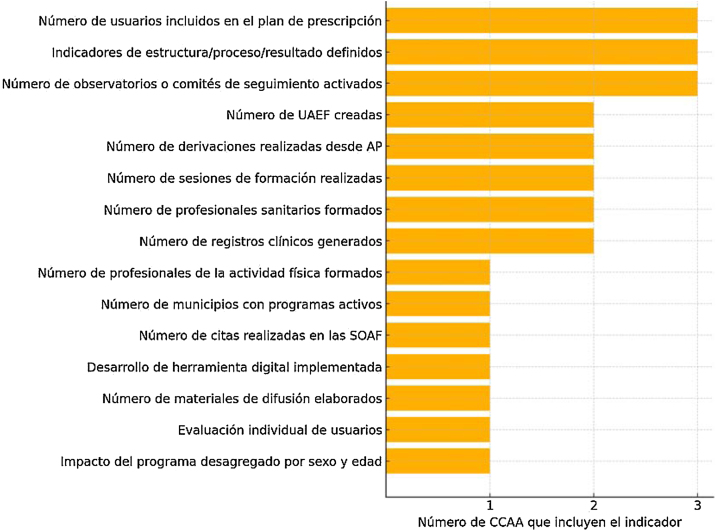
Indicadores comunes en los planes autonómicos de prescripción de actividad física y ejercicio (PPAFyE).

En la figura 3 se muestra un resumen de los indicadores comunes de evaluación y el número de CCAA en las que se encuentran presente.

## Discusión

El objetivo principal de este estudio fue analizar la existencia en las CCAA de PPAFyE, comparando e identificando tipos de medidas propuestas, poniendo en valor el contexto de la AP, el personal de enfermería como figura prescriptora y el uso de activos en salud y recursos comunitarios. Los resultados muestran que 11 CCAA cuentan con PPAFyE y las que no tienen son Asturias, Aragón, Castilla la Mancha, Castilla León, La Rioja, Murcia y las ciudades autonómicas de Ceuta y Melilla. Se desconocen las razones a pesar de existir una resolución de 4 de julio de 2022, de la Presidencia del Consejo Superior de Deportes, publicado en el BOE 171/2022 de 18 de julio, que entre sus objetivos esta disponer en cada CCAA de un sistema de prescripción de actividad y ejercicio físico eficiente. Por otro lado, el compromiso institucional de Andalucía, Cantabria, Extremadura, País Vasco y Comunidad Valenciana se refleja en la publicación oficial de sus planes en los respectivos Boletines Oficiales, consolidando así estas estrategias como políticas públicas de salud. Asturias y Baleares han promulgado recientemente leyes autonómicas (Ley 5/2022 y Ley 2/2023, respectivamente) que reconocen la actividad física y el deporte como un derecho ciudadano, aunque sin integrar de forma clara a los sistemas sanitarios ni a profesionales de la salud en un modelo estructurado de prescripción. Por su parte, Navarra y Comunidad Valenciana contemplan la prescripción desde AP, pero sin especificar qué perfiles profesionales la ejercen, lo que evidencia una falta de homogeneidad. Cataluña, pese a contar con un plan específico, no incluye la AP ni define agentes prescriptores. Sin embargo, se ha demostrado que los profesionales de AP, especialmente enfermería, son clave en la educación para la salud y en mejorar indicadores mediante prescripción de AFyE^15^, ^16^, ^17^, ^18^.

La función prescriptora no se limita exclusivamente al ámbito sanitario, sino que contempla la derivación a UAEF gestionadas por profesionales CAFyD. Este enfoque interprofesional resulta esencial para reducir brechas, fomentar alianzas y, sobre todo, generar confianza entre los equipos de AP y los profesionales encargados de implementar las intervenciones en centros deportivos o comunitarios, facilitando así la integración del ejercicio como herramienta terapéutica^19^. Un modelo basado en equipos interdisciplinarios favorecería una mayor accesibilidad, continuidad asistencial y un abordaje más integral de los determinantes sociales y conductuales asociados al sedentarismo.

Nueve PPAFyE analizados incluyen acciones formativas dirigidas tanto a profesionales sanitarios como a profesionales CAFyD, lo que refleja un enfoque intersectorial y una intención clara de dotar de competencias a los responsables de implementar estas estrategias. Esta necesidad formativa está avalada por estudios que evidencian carencias en el conocimiento sobre la prescripción de ejercicio en AP, especialmente en pacientes con enfermedades crónicas o condiciones clínicas complejas^20^.

Solo cuatro PPAFyE incluyen medidas de investigación. Destaca el caso de Extremadura, que en 2021 estableció un convenio con la Universidad de Extremadura a través del «Observatorio El Ejercicio te Cuida»^21^, aunque aún no se dispone de publicaciones formalmente atribuidas a dicho observatorio. Ocho PPAFyE incluyen indicadores de evaluación, lo que permite medir con precisión resultados operativos y económicos, mejorar los programas y facilitar la rendición de cuentas^22^. Como señalan Ignacio et al. (2025), una evaluación estructurada permite valorar objetivamente su calidad y eficacia mediante estándares claros^23^. En cambio, los planes sin indicadores dependen de valoraciones subjetivas, limitando su capacidad de adaptación a las necesidades reales de la población.

## Limitaciones del estudio

Este estudio presenta varias limitaciones que deben considerarse al interpretar los resultados. La revisión documental se realizó en junio de 2025, por lo que algunos planes autonómicos podrían haberse publicado posteriormente, afectando la exhaustividad del análisis. No obstante, se aplicaron criterios explícitos de inclusión que garantizan la validez del diagnóstico autonómico en ese momento. Además, no se analizaron iniciativas impulsadas por ayuntamientos, mancomunidades u otras entidades locales, ni programas del Ministerio de Sanidad u otros organismos estatales. Esta exclusión responde al enfoque metodológico centrado exclusivamente en los planes elaborados por las comunidades autónomas en el ámbito de la AP, dado su carácter competencial en materia de salud. Aunque estas iniciativas podrían ser complementarias, su análisis requeriría otro diseño de investigación. Por último, los resultados reflejan una marcada heterogeneidad en la implementación autonómica, lo que evidencia la necesidad de avanzar hacia un modelo nacional más coordinado, con criterios comunes y mecanismos sistemáticos de evaluación.

## Conclusión

Once Comunidades Autónomas en España, han desarrollado planes o programas de prescripción de actividad física y ejercicio, la mayoría reconociendo a la Atención Primaria como contexto idóneo y al personal de enfermería como figura prescriptora clave. Sin embargo, existe una importante heterogeneidad territorial, lo que limita el acceso equitativo de la población a esta estrategia de salud. La incorporación de recursos comunitarios y activos en salud al proceso de prescripción promueve la colaboración intersectorial con los ámbitos sanitario, deportivo, educativo y municipal.

**Lo conocido sobre el tema**

• La prescripción de actividad física y ejercicio en Atención Primaria es clave frente al sedentarismo.
• Existen iniciativas autonómicas, sobre planes de prescripción, pero su desarrollo es heterogéneo.

**Qué aporta este estudio**

• Mapea los planes de prescripción de actividad física y ejercicio en España y documenta su implementación (11/17): 10 incluyen la Atención Primaria y todos integran recursos comunitarios.
• Cuantifica el papel de enfermería (8/11) y la presencia de evaluación con indicadores (8/11) e investigación (4/11).
• Identifica brechas y propone avanzar hacia indicadores comunes y coordinación intersectorial.

## Financiación

Ninguna.

## Consideraciones éticas

En el presente estudio no estuvieron involucradas personas participantes, siendo un análisis documental de dominio público.

## Declaración de la IA generativa y tecnologías asistidas por IA en el proceso de escritura

Durante la preparación de este manuscrito, los autores utilizaron ChatGPT (OpenAI) exclusivamente para apoyo lingüístico y de estilo, homogeneización de referencias/DOI y formatos bibliográficos y borradores iniciales de textos estandarizables. La herramienta no se empleó para el análisis de datos, extracción de resultados, elaboración de discusión, conclusiones científicas ni generación de contenido original que sustituyera el juicio experto. Las indicaciones proporcionadas a la herramienta no incluyeron datos sensibles. Tras su uso, todos los contenidos fueron revisados críticamente, verificados y editados por los autores, quienes asumen la plena responsabilidad de la exactitud, integridad y originalidad del manuscrito. De acuerdo con las políticas editoriales, la IA no se considera autora ni cumple criterios de autoría.

## Conflicto de intereses

Los autores declaran no tener ningún conflicto de intereses.
